# The “Love Hormone” Oxytocin Regulates the Loss and Gain of the Fat–Bone Relationship

**DOI:** 10.3389/fendo.2015.00079

**Published:** 2015-05-18

**Authors:** Graziana Colaianni, Li Sun, Mone Zaidi, Alberta Zallone

**Affiliations:** ^1^Department of Basic Medical Science, Neuroscience and Sense Organs, University of Bari, Bari, Italy; ^2^Mount Sinai Bone Program, Mount Sinai School of Medicine, New York, NY, USA

**Keywords:** oxytocin, bone, fat, muscle, hormone

## Abstract

The involvement of oxytocin (OT) in bone metabolism is an interesting area of research that recently achieved remarkable results. Moreover, several lines of evidence have largely demonstrated that OT also participates in the regulation of energy metabolism. Hence, it has recently been determined that the posterior pituitary hormone OT directly regulates bone mass: mice lacking OT or OT receptor display severe osteopenia, caused by impaired bone formation. OT administration normalizes ovariectomy-induced osteopenia, bone marrow adiposity, body weight, and intra-abdominal fat depots in mice. This effect is mediated through inhibition of adipocyte precursor differentiation and reduction of adipocyte size. The exquisite role of OT in regulating the bone–fat connection adds another milestone to the biological evidence supporting the existence of a tight relationship between the adipose tissue and the skeleton.

## Introduction

Aging is associated with high incidence to develop both obesity and osteoporosis (1), which are often simultaneous pathological conditions deriving from an altered balance between fat and bone cells in bone marrow. During menopause period, the onset or worsening of obesity and osteoporosis dramatically occurs. Several pharmacologic strategies exert opposite effects on fat versus bone mass. For instance, the sex hormone replacement treatment has proved to be effective in mitigating bone loss (2, 3) and reversing menopause-related obesity (4). Likewise, but with opposite effect, therapy with glucocorticoids could affect bone remodeling (5–7) and increase obesity (8) or bone marrow infiltration by adipocytes (1). The ongoing studies on the balance of adipose and bone cell differentiation in bone marrow have clearly established a negative association between fat and bone mass. Adipocytes and osteoblasts originate from a common mesenchymal precursor that can also differentiate into other cell types, but among the various fates, differentiation of adipocyte or osteoblast becomes of particular relevance because factors that enable osteoblastogenesis inhibit adipogenesis and *vice versa*.

Recent studies have grown the interest on pituitary hormones as endocrine skeletal regulators, demonstrating that their levels correlate with bone microstructure and bone turnover markers during menopause transition (9). In fact, the pituitary–bone axis importance is widely acknowledged, demonstrating that several pituitary hormones, such as growth hormone (GH) (10), follicle stimulating hormone (FSH) (11), thyroid stimulating hormone (TSH) (12), prolactin (PRL) (13), oxytocin (OT) (14), and vasopressin (15) regulate skeletal homeostasis. Likewise, haploinsufficient mice for pituitary hormones or their receptors showed severe skeleton defects while the primary target organ could remain unaffected, indicating that the skeleton is more responsible to the pituitary hormone control (10–15). Remarkably, haploinsufficient OT^+/−^ or OTR^+/−^ mice, while lactating normally, exhibit profound osteopenia (14). In humans, plasma OT levels directly correlate with the development of osteopenia or osteoporosis in postmenopausal women, as demonstrated by Breuil et al. (16, 17). In their studies, authors showed that elevated OT levels are associated with high-bone mineral density (BMD), in particular, at the hip of women with low estradiol or high-leptin serum levels (16). Likewise, osteoporotic patients with low OT serum levels display severe osteoporosis (17), and this association is independent from other factors regulating OT serum levels, such as estradiol or leptin (17).

Furthermore, several reports demonstrated that adipocytes express OT receptors (OTRs) (18, 19). Elabd et al. (19) identified OTR as a potential regulator of the osteoblast/adipocyte balance in human multipotent adipose-derived stem (hMADS) cells. Both OT and carbetocin (a stable OT analog) negatively modulated adipogenesis while promoting osteogenesis in both hMADS cells and human bone marrow mesenchymal stromal cells (19).

Notably, another recent study of Elabd and colleagues (20) revealed that OT acts directly on muscle stem cells, exerting a pro-myogenic effects mediated by MAPK/ERK signaling. Additionally, authors reported that plasma OT levels and OTR expression in muscle stem cells dramatically decline during aging, demonstrating that OT has a pivotal role also in homeostasis of skeletal muscle tissue (20).

The main goal of researches on OT physiology has been to gather new insights in the bone–fat–muscle connection, in order to prevent and treat diseases related to the altered communication among these tissues. For instance, during aging, the lack of mobility due to muscle mass decline could exacerbate obesity and/or osteoporosis and *vice versa*. On the other hand, obesity also leads to metabolic disorders, which in turn stimulates muscle wasting and bone loss. Although the therapeutic potential of OT to treat musculoskeletal and metabolic conditions in humans remains to be determined, data from OT deficient rodent models have revealed encouraging results (Table 1).

**Table 1:** **Mice phenotype**.

	OT^−/−^OTR^−/−^mice (male and female)
Bone tissue phenotype	Osteopenic (at the birth; worsen with age)
Adipose tissue phenotype	Obese (late-onset)
Muscle tissue phenotype	Sarcopenic (early-onset)

## Peripheral OT Synthesis in Bone Marrow is Estrogen Mediated

Estrogen dependence of OT and its receptors synthesis (21–23) further broadened the importance of OT/OTR biological role. OT is locally synthesized in several organs (24–26) and, like in other tissues, estrogen stimulates OT production in bone (23). 17β-estradiol stimulates OT production in osteoblasts through Erk phosphorylation, following a non-classical, ERE-independent pathway. The binding of 17β-estradiol to cell membrane estrogen receptors (ERs), rather than nuclear ERs, is required to induce OT synthesis. Thus, the relatively cell-impermeant analog 17β-estradiol–BSA conjugate has shown to be effective in stimulating Erk1/2 phosphorylation within 3 min and OT expression within 2 h (23). On the contrary, native 17β-estradiol, but not the estradiol–BSA conjugate, increased OTR expression at ~6 h, indicating that OTR induction by estrogen occurs through a traditional genomic mechanism (23). This local circuit of OT produced in bone, in response to estrogen, acts upon OTR to stimulate further OT release, which enhances estrogen action (23).

## OT Directly Regulates Bone Homeostasis

The maximal fetal and post-natal bone growth occurs during the last phase of pregnancy and lactation when the mother loses ~120 g of calcium from her skeleton (27). During the intergenerational calcium transfer, the fetal skeleton is mineralized at the expense of the mother. This bone loss was quantified as 1–3% per month, thus much higher than 1–3% of bone loss per year affecting women with postmenopausal osteoporosis (28). Experimental evidence, demonstrating a direct action of OT on the skeleton during pregnancy and lactation, revealed that this hormone plays an important role in orchestrating the intergenerational calcium transfer (29). Pups from mothers with genetic OT-deficiency displayed apparently normal skeletons, without bone/cartilage defects and any difference in bone volume fraction (BV/TV) of trabecular bone. Instead, increase in trabecular number, decrease in trabecular spacing, and no change in trabecular thickness were found (29). However, consisting with the unchanged BV/TV, even in the face of greater trabecular number, this result suggested that in OT^−/−^ pups each trabecula was less mineralized than in wild type (29).

The OT action on the skeleton is mainly mediated not only through its stimulation of osteoblast differentiation but also through a modulation of osteoclast formation and function. OT and OTR knock out mice develop low turnover osteoporosis that worsens with age in both genders (2). Bone assessment analysis revealed a pronounced decrease in vertebral and femoral trabecular volume, already evident in the haploinsufficient mice, which is accompanied by a significant reduction in bone formation rate (14). *Ex vivo* osteoblasts from OT^−/−^ and OTR^−/−^ mice had a lower expression of all master genes involved in osteoblast differentiation and produced fewer mineralized nodules than osteoblast from wild-type littermates. Treatment with recombinant OT led to up-regulation of bone morphogenetic protein 2 (Bmp-2) and activating transriptor factor 4 (Atf-4), inducing osteoblast development toward a mineralizing phenotype (14). At the same time, OT stimulated osteoclast differentiation by increasing ratio of receptor activator of nuclear factor-kappaB ligand (RANK-L) and osteoprotegerin (OPG), while inhibited bone resorption by triggering cytosolic Ca^2+^ release and nitric oxide synthesis (14). The skeletal action of OT is mediated by OTR internalization and its subsequent translocation to the nucleus through β-arrestin (Arrb). In osteoblasts, OTR interacts with Rab5 and then binds to the karyopherin transportin-1 (Tnpo1), which facilitates nuclear transport. OTR intracellular trafficking to the nucleus is abolished knocking down Arrb or Tnpo1 and, consequently, the action of OT on osteoblast differentiation genes, namely osterix, Atf-4, bone sialoprotein, and osteocalcin is dramatically abrogated (30). As other G protein-coupled receptors (GPCRs), OTR internalization can activate signaling pathways quite distinct from those activated by the same receptors on the cell surface. Notably, effects of OT on osteoblast differentiation, exerted through its nuclear translocation, are independent of its effects on activating the MAPK pathway. In fact, despite knocking down Arrb isoforms, which abrogate the effects of OTR on gene expression, the pErk signal remained unaffected (30).

## OT Reverses Ovariectomy-Induced Gain of Fat Mass

Several lines of evidence demonstrated that OT signal and energy homeostasis are often correlated (31, 32). Following food intake, a peak of circulating OT levels across a 24-h period was found in mice (31) and hypothalamic OT mRNA expression was decreased with fasting and, subsequently, recovered upon refeeding (32). Noteworthy, the hyperphagic obesity of single-minded 1 (*Sim1*) haploinsufficient mice has been explained by reduced OT expression (33), whose obese phenotype can be rescued with OT treatment (33). Human patients suffering hyperphagia and obesity, such as those affected by *SIM1* gene mutation (34) and by Prader–Willi syndrome (35), have reduced number and size of OT neurons in the paraventricular nucleus of the hypothalamus. However, OT can also affect body mass independently from reduction of food intake. Accordingly, OTR null mice develop late-onset obesity characterized by augmentation of abdominal fat pads, even in the face of unchanged daily intake of chow (36). Data from Elabd et al. showed that hypogonadal-induced bone loss and fat mass increase were both linked to low OT circulating levels, and that restoring OT levels could therefore reverse osteopenia and fat mass increase in OVX mice, used as animal model mimicking the menopause (19).

It is well known that OVX mice gain body weight after surgery and exhibit increased intra-abdominal fat mass. OVX mice treated with OT displayed a significantly reduction in body weight compared with mice treated with vehicle. Interestingly, OT did not significantly affect the body weight of Sham operated mice. Of note, the OT treatment was less effective in decreasing body weight compared to the quite higher effects on bone parameters (37). Furthermore, plasma and liver triglyceride as well as glucose tolerance were not affected by the OT treatment. Interestingly, consistent with the normalization of body fat mass, insulin secretion was normal, suggesting that OT might protect against ovariectomy-induced insulin resistance. Circulating levels of osteocalcin and its undercarboxylated form were not altered following OT treatment after ovariectomy, thus indicating that osteocalcin is not involved in OT signaling in adipose tissue (37). Finally, the observations that OT treatment did not significantly reduce adipocyte size, in ovariectomy-induced hypertrophic fat depots, suggest that OT led to a reduction of fat mass mainly through a decrease in the formation of new adipocytes rather than a decrease of adipocyte hypertrophy (37).

## Conclusion

Menopause, characterized by high-skeletal fragility and often accompanied by increased adiposity, is a worldwide heavy burden for public health. Osteoblasts and adipocytes, the building cells of bone and adipose tissue, respectively, share a common origin and, throughout life, feature an inverse relationship in their differentiation processes. Therefore, the development of new therapeutic strategies for treating osteoporosis and obesity might require identifying signaling pathways that stimulate mesenchymal stem cells toward osteogenesis at the expense of adipogenesis. In view of a potential role of OT to shift the balance in favor of osteogenesis and against adipogenesis, further investigations will be relevant to determine if results obtained in rodents can be translated in human subjects. Considering that OT is an FDA-approved drug, it would be extremely useful as skeletal anabolic agent to treat osteoporosis, but might also have potential utility in treating obesity and adipose tissue-related pathologies. Noteworthy, recent findings demonstrating that OT is required for muscle tissue regeneration and homeostasis, suggested a novel potential way to prevent sarcopenia, which often accompanies osteoporosis and obesity during aging.

## Conflict of Interest Statement

The authors declare that the research was conducted in the absence of any commercial or financial relationships that could be construed as a potential conflict of interest.
